# Effect of Molecular Structure in the Chain Mobility of Dichalcogenide-Based Polymers with Self-Healing Capacity

**DOI:** 10.3390/polym11121960

**Published:** 2019-11-29

**Authors:** Mikel Irigoyen, Jon M. Matxain, Fernando Ruipérez

**Affiliations:** 1 POLYMAT, University of the Basque Country UPV/EHU, Joxe Mari Korta Center. Avda. Tolosa 72, 20018 Donostia - San Sebastián, Spain; mikel.irigoyen@polymat.eu; 2 Kimika Fakultatea, Euskal Herriko Unibertsitatea UPV/EHU and Donostia International Physics Center (DIPC), P.K. 1072, 20080 Donostia, Spain

**Keywords:** self-healing polymers, molecular dynamics, non-covalent interactions

## Abstract

Recently, it has been shown that the reaction mechanism in self-healing diphenyl dichalcogenide-based polymers involves the formation of sulfenyl and selenyl radicals. These radicals are able to attack a neighbouring dichalcogenide bond via a three-membered transition state, leading to the interchange of chalcogen atoms. Hence, the chain mobility is crucial for the exchange reaction to take place. In this work, molecular dynamics simulations have been performed in a set of disulfide- and diselenide-based materials to analyze the effect of the molecular structure in the chain mobility. First of all, a validation of the computational protocol has been carried out, and different simulation parameters like initial guess, length of the molecular chains, size of the simulation box and simulation time, have been evaluated. This protocol has been used to study the chain mobility and also the self-healing capacity, which depends on the probability to generate radicals (ρ), the barrier of the exchange reaction (ΔG) and the mobility of the chains (ω). The first two parameters have been obtained in previous quantum chemical calculations on the systems under study in this work. After analyzing the self-healing capacity, it is concluded that aromatic diselenides (PD-SeSe) are the best candidates among those studied to show self-healing, due to lower reaction barriers and larger ω values.

## 1. Introduction

The concept of dynamic covalent chemistry, where certain covalent bonds may undergo reversible cleavage and reformation, was introduced by Rowan and coworkers [1]. This feature can be incorporated in polymeric materials to introduce new functionalities, leading to stimuli-responsive materials [2,3,4,5] with new characteristics, such as property regulation, reprocessing and self-healing capacity, for instance [6,7,8,9]. The dynamic character can be achieved by means of non-covalent interactions like hydrogen bonds [10], π-π stacking [11] or metal-ion interactions [12], but the use of dynamic covalent bonds has increased in the last few years, as its use ensures both dynamic behavior and robustness [13,14,15,16]. With this purpose, several chemistries have been used such as the Diels-Alder reaction [17,18], siloxane exchange [19,20], transesterification [21,22] or disulfides [23,24].

The disulfide bond has been widely used in the synthesis of self-healing materials due to its reversibility [25,26,27,28,29,30], although external stimuli such as catalysts [26,31,32], irradiation [25,27,30,33] or high temperature [34,35] are often needed to trigger the self-healing exchange reaction. Nevertheless, it has been found that this reaction occurs at room temperature for aromatic disulfides and their incorporation has successfully produced self-healing elastomers, epoxy networks and polyurethanes [36,37,38,39,40,41,42]. Other dichalcogenides such as diselenides or mixed sulfur-selenium bonds show the same dynamic character as disulfides [43,44] and they have been used recently to produce new self-healing materials [45,46]. Similar to disulfides, aromatic diselenides have been found, both theoretically [47] and experimentally [48], to exchange at room temperature without the need of external stimuli, even faster than their disulfide counterparts, and, as for disulfides, aromatic diselenides show a more efficient self-healing procces than the aliphatic ones [49].

The mechanism responsible of the self-healing reaction in dichalcogenide-based polymers has been studied computationally by Matxain, Ruipérez and collaborators [50]. These authors proposed a [2+1] radical-mediated mechanism to be responsible for the self-healing reaction, a fact that was later confirmed experimentally in the absence of any catalyst [51]. In this manner, the first step of the exchange reaction is the cleavage of the dichalcogenide bond, and thus, the generation of chalcogenyl radicals, which may then be able to attack another neighbouring dichalcogenide bond. In the process, a three-membered transition state is created, producing a new radical in a single-displacement reaction and another dichalcogenide bond, leading to the interchange of chalcogen atoms in the process. Based on this, a computational protocol to assess the theoretical self-healing capacity in these materials was proposed using three parameters [52]: (i) the probability to generate chalcogenyl radicals (ρ) calculated from quantum molecular dynamics, which is governed by the dissociation of the dichalcogenide bond and, to less extent, by the hydrogen bonding between chains, (ii) the exchange reaction rate constant (*k*), calculated by Transition State Theory, and (iii) the mobility of the chains (ω), estimated as the probability of finding two chalcogenide bonds close enough to react. This last parameter is largely governed by non-covalent interactions and is defined as the number of dichalcogenides that lie inside the so-called reacting region, where dichalcogenides are close enough to undergo the exchange reaction.

In previous works [47,50,53], both aliphatic and aromatic disulfide- and diselenide-based materials, including different substituents, have been studied by analyzing the parameters obtained from quantum chemical calculations, namely, ρ and *k*. Only in the work of Formoso et al. [52] the effect of the chain mobility (ω) of some aromatic disulfide-containing urea and urethanes was estimated by means of molecular dynamics. It was observed that hydrogen bonding was the most relevant feature affecting chain mobility and that both a large and a small number of hydrogen bonds were detrimental for the self-healing process, as chains would be either too rigid, avoiding the contact between disulfide bonds, or too mobile, decreasing the number of disulfides in the reaction region (lower values of ω). In this way, systems with intermediate number of hydrogen bonds showed larger values of ω.

**Figure 1:**
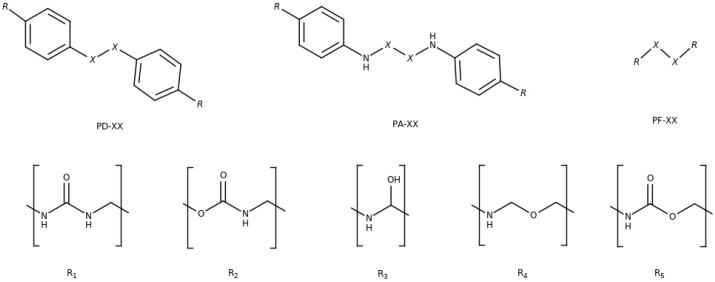
Disulfides (X = S) and diselenides (X = Se) studied in this work. **Top**: diphenyl (PD), diphenyl amino (PA) and phenyl free (PF) backbones. **Bottom**: the five substituents, urea (R${}_{1}$), urethane (R${}_{2}$), secondary alcohol (R${}_{3}$), ether (R${}_{4}$) and ester (R${}_{5}$).

Therefore, in order to complete these previous works and to provide with a full theoretical self-healing capacity, in this work we perform a study of ω in the molecular systems for which ρ and *k* parameters have been previously analyzed. In this way, we aim to investigate the effect of the molecular structure in the non-covalent interactions and in the mobility of the chains. Concretely, diphenyl (PD-XX), diphenyl amino (PA-XX) and phenyl free (PF-XX) dichalcogenides have been studied, where X stands for S and Se (see Figure 1). Five substituents (R${}_{n}$) have been considered with the aim of resembling different polymeric chains, where these PD-XX, PA-XX and PF-XX cross-linkers may be inserted. Thus, the studied systems are labeled as R${}_{n}$-YY-XX, where n = 1–5, YY = PA, PD, PF and XX = S-S, Se-Se. Besides, the reliability of the molecular models used by Formoso [52] will also be assessed and a validation of the computational protocol will be performed.

## 2. Methods

### 2.1. Molecular Dynamics Simulations

The molecular dynamics (MD) simulations have been performed using ff14SB [54] amber force-field in the AMBER 14 molecular dynamics simulation package [55]. The parameters corresponding to the diphenyl diselenide derivatives have been obtained by Torsello et al. [56]. All structures were built via the LEaP module of Ambertools and the charges were computed using the restrained electrostatic potential (RESP) fitting procedure [57]. First the ESP was calculated by means of the Gaussian package [58] using the 6-31G* basis set at Hartree-Fock level of theory and then the RESP charges were obtained. All the simulations were carried out in vacuum in a canonical ensemble (NVT) with a 2 fs timestep. 100,000 steps of minimization (50,000 steps steep descent minimization plus 50,000 steps of conjugate gradient minization) were followed by heating from 80 to 300 K over 200 ps, an equilibration time of 1800 ps with a Langevin thermostat and a production of 30 ns for the polymeric chains (between 1456 and 1808 atoms). Covalent bond lengths involving hydrogen were constrained using the SHAKE algorithm. The initial random conformations of the molecular systems have been generated using Packmol package [59].

In order to analyze the hydrogen bonds, we consider a threshold for the bond length between the donor and acceptor (X⋯Y) smaller than 3.5 Å and the range of X-H⋯Y angles varies between 140 and 220 degrees. In addition, to see the evolution of the hydrogen bonding along the simulation time, the hydrogen-bond occupancy is defined as $HB_{Occ}=100\frac{\sum nHB}{t}$, where nHB denotes the number of formed hydrogen bonds, at a given step, according to the criteria for the bond lengths and angles mentioned above, and *t* is the number of simulation frames. Thus, $HB_{Occ}$ represents the fraction of time that the hydrogen bond is formed. In addition to this, in order to describe the size of a polymer chain, the radius of gyration ($r_{gyr}$) is defined as $r_{gyr}^{2}=\frac{\left(\sum_{i=1}^{N}{\left(r\left(i\right)-\bar{r}\right)}^{2}\right)}{N}$, where $\bar{r}$ is the geometric center of mass of the system. It corresponds to the root-mean-square distance of the atoms from the center of mass.

### 2.2. Validation of the Computational Protocol

In the work of Formoso et al. [52], molecular dynamics calculations were performed using certain simulation constrains, such as the size of the molecular model or the simulation time, which could be too restrictive. In this study, with the aim of using more realistic conditions, different simulation parameters have been evaluated to see their influence in the non-covalent interactions among chains. In particular, we have tested the following: (i) the initial molecular guess, (ii) the length of the chains, (iii) the size of the simulation box and (iv) the simulation time. In order to do such analysis, the R${}_{1}$-PD-SS and R${}_{2}$-PD-SS (urea- and urethane-containing diphenyl disulfides) molecular systems have been chosen. For the validation, we make use of the number of hydrogen bonds and their impact in ω. This parameter is based on the distance between dichalcogenides and is defined as [52]:

(1) $$\omega =\frac{I_{i}}{I_{i}+I_{ii}}$$

Using the radial distribution function of the S or Se atoms, three regions may be defined to calculate ω: the reacting region ($I_{i}$), where dichalcogenides are close enough to undergo the exchange reaction (R ≤ 4.5 Å), the neighboring region ($I_{ii}$), where dichalcogenides are far to react but with a non-negligible probability to approach the reacting region (4.5 < R < 20 Å), and the external region ($I_{iii}$), where dichalcogenides are neglected, R > 20 Å. Note that the external region is not included in the definition of ω in order to have a size-independent parameter. The amount of chalcogen atoms located in each region is calculated by integration of the radial distribution function within the limits defined above. These limits are defined for disulfide compounds and will be different for diselenides, as it will be discussed hereafter.

#### 2.2.1. Initial Guess and Chain Length

**Figure 2:**
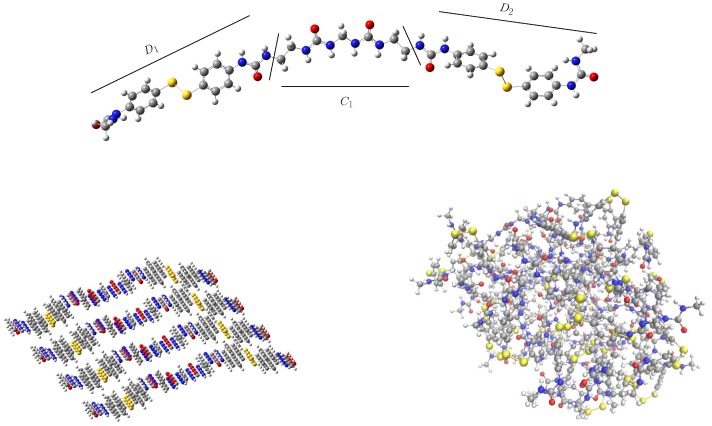
**Top**: Single chain of the molecular model used in the validation step, consisting of two urea-substituted diphenyl disulfides (D${}_{1}$ and D${}_{2}$ units) linked by a polyurea-like chain (C${}_{1}$ unit). **Bottom**: periodic (**left**) and random (**right**) initial conformations before equilibration. Sulfur atoms in yellow, nitrogen in blue and oxygen in red.

In the previous work, the model comprised a single chain formed by two phenyl disulfide monomers (D${}_{1}$ and D${}_{2}$ units in Figure 2, top) linked by a urea-methyl-urea or urethane-methyl-urethane unit (C${}_{1}$ in the same Figure). This chain was then replicated in space in order to generate a 3D structure. For that, four replicas of the chain were placed along two spatial directions using translation vectors. The resulting initial conformation, before the equilibration step, is depicted in Figure 2 (bottom left). It can be observed that the use of translation vectors yields a laminar-like periodic structure that may introduce a structural bias. In this work, we have considered a random initial guess (Figure 2, bottom right) in order to have a more realistic description of a complex polymeric system. Besides, the chain length has also been increased by including one additional –(CH${}_{2}$)– group between both disulfides and the polyurea chain (C${}_{1}$ unit).

The results of the simulations for the two initial structures (periodic and random) and the two molecular chains (with and without the additional methyl group, labeled as long and short, respectively) are collected in Table 1, where the total number of hydrogen bonds (HB${}_{tot}$) and the maximum (HB${}_{max}$), minimum (HB${}_{min}$) and average (HB${}_{ave}$) number of hydrogen bonds at a given step of the simulation are provided, together with ω. Besides, two simulation times (10 and 30 ns) are considered, that will be discussed later.

**Figure 3:**
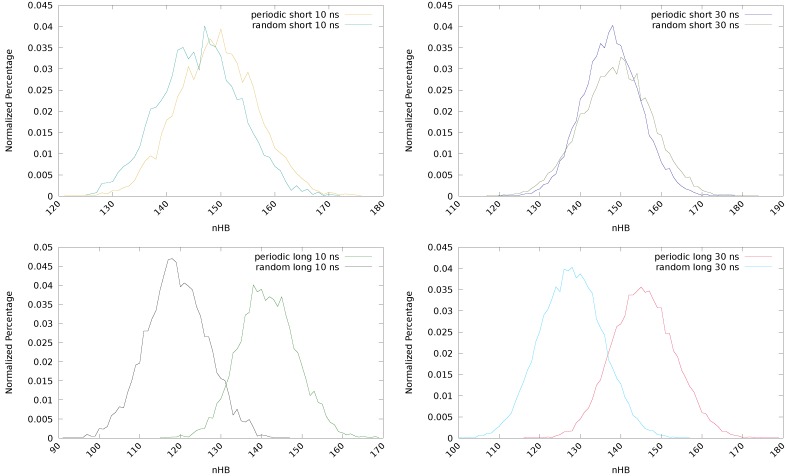
Normalized percentage of hydrogen bonds of the periodic and random conformations with short (**top**) and long (**bottom**) chains, obtained after 10 (**left**) and 30 (**right**) ns of simulation.

Considering the initial guess for the short chain, the total number of hydrogen bonds is similar for both systems but the distribution is different. See Table 1 and also Figure 3 (top), where the normalized percentage of hydrogen bonds is represented. In the periodic structure, the hydrogen bonds are mainly formed between the same components of different chains, that is, both the disulfide units and the linking chain tend to form HBs with their counterparts in the surrounding chains (D${}_{1}$-D${}_{1}$, D${}_{2}$-D${}_{2}$ and C${}_{1}$-C${}_{1}$), see Figure 2, bottom left. This introduces a bias that will affect the equilibration step and, therefore, the rest of the simulations. On the other hand, in the random conformation, each unit can establish HBs with any other unit of the surrounding chains that is close enough, and may be considered as a more realistic situation. This change in the distribution is responsible of the observed decrease in ω for the random conformation. In the periodic model, disulfide bonds are located periodically along the system, favoring the D${}_{1}$-D${}_{1}$ and D${}_{2}$-D${}_{2}$ interactions and maximizing the value of ω. On the other hand, the interactions between disulfides laying on different sides of the chain (D${}_{1}$-D${}_{2}$) will be more scarce, contributing to a lowering of this parameter. In the random conformation, however, an opposite situation may take place. Since the disulfides will not necessarily have another disulfide nearby, the probability of the D${}_{i}$-D${}_{i}$ interaction is lower and, hence, ω will also be smaller. Nevertheless, the probability of the D${}_{i}$-D${}_{j}$ interaction is higher, contributing to enlarge ω. The net effect, as it is observed in Table 1, is that ω is lower for the random conformation. This effect is found to be independent of the length of the chain and the simulation time. This means that the use of a periodic model induces an arbitrary preference in the interaction between adjacent disulfides (D${}_{i}$-D${}_{i}$) and ω may be overestimated.

If a long chain is considered (with an extra –(CH${}_{2}$)– group), notable differences are found between the periodic and the random systems regarding the hydrogen bonds. In both cases, a decrease of the total number of hydrogen bonds is observed, but it is particularly pronounced for the random system (from 730,119 to 595,587, after 10 ns). A larger chain unit (C${}_{1}$) means a separation of the groups that can form HBs, both intermolecular and intramolecular, reducing the possibility of interactions and, therefore, the total (HB${}_{tot}$), maximum (HB${}_{max}$) and average (HB${}_{ave}$) number of hydrogen bonds are decreased. In the periodic system, this effect is small, since the laminar structure is kept and the starting HBs (from the D${}_{i}$-D${}_{i}$ and C${}_{1}$-C${}_{1}$ interactions) remain. The observed reduction (from 746,461 to 707,860, after 10 ns) may be ascribed to the changes in the geometry of the chains induced by the new methyl group. In fact, this effect is largely mitigated in the longer (30 ns) simulation of the periodic structure, since a longer simulation time allows the structure to reorganize and recover the lost HBs. The differences between the periodic and random structures are clearly observed in Figure 3 (bottom), in the normalized percentage of hydrogen bonds after 10 ns (left) and 30 ns (right) of simulation time.

**Figure 4:**
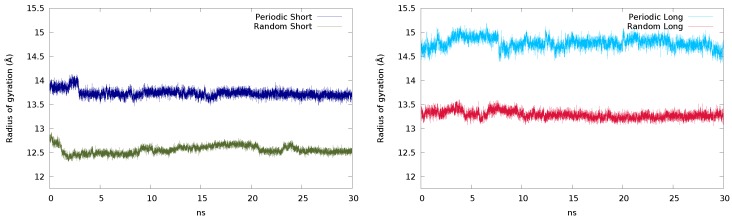
Radius of gyration (Å) of the periodic and random systems with the short (**left**) and long (**right**) chains obtained in 30 ns simulations.

Comparing the radius of gyration of the short chains (Figure 4, left), the periodic system oscillates close to 14 Å, while the one corresponding to the random system appears around 12.5 Å. This suggests that the periodic structure somewhat retains the laminar-like structure after the equilibration, while the random structure acquires a more coiled conformation. When the system is built with long chains, the same feature is observed (Figure 4, right), but with larger values.

As a summary, we can conclude that the periodic starting structure is less suitable to perform the simulations, since its periodicity introduces a bias that may affect to the estimation of ω.

#### 2.2.2. Size of the Simulation Box

**Figure 5:**
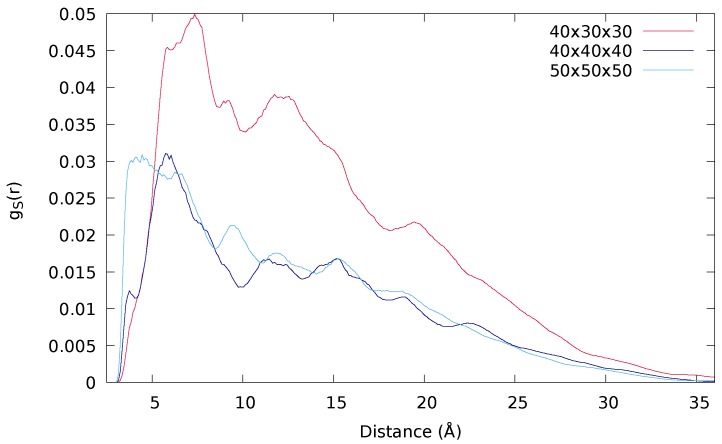
Radial distribution functions of the PD-urea derivative including the extra –(CH${}_{2}$)– obtained in 10 and 30 ns simulations by using simulation boxes of 40 × 30 × 30 (red line), 40 × 40 × 40 (dark blue line) and 50 × 50 × 50 nm (light blue line).

Another important parameter is the size and shape of the simulation box, which determines the density of the system. In the simulations of Ref. [52], a square prism of 40 × 30 × 30 nm was used and now it has been compared to a cubic box of two different sizes: 40 × 40 × 40 nm and 50 × 50 × 50 nm. The results are collected in Table 2 and Figure 5, where the radial distribution function of each sulfur with respect to the others is represented, excluding their counterparts in the disulfide S-S bond. Despite the number of hydrogen bonds remains basically unchanged, the shape and the size of the box have a remarkable impact on the radial distribution function and, thus, in ω. This suggests that there is a frontier effect that may be avoided using larger and regular boxes.

In conclusion, having all these considerations in mind, a random conformation with long chain have been selected to carry out the molecular dynamics simulations, using a cubic simulation box of 50 × 50 × 50 nm, with a simulation time of 30 ns for each system, given that the longer the simulation time allows the exploration of a bigger number of conformations.

## 3. Results

Once the simulation conditions have been optimized, the results obtained for all the R${}_{n}$-YY-XX molecular systems are presented and analyzed. In particular, we discuss the influence of the backbone YY (PD, PA, PF), substituents R${}_{n}$ (*n* = 1–5) and chalcogenide XX (X = S, Se) in: (i) the dimension and flexibility of the chains, (ii) the non-covalent interactions, namely, the hydrogen bonding, and (iii) the radial distribution functions of the sulfur and selenium, from where ω is obtained. Finally, we will estimate the self-healing theoretical capacity for each material.

### 3.1. Influence of the Backbone: PD, PA And PF

**Figure 6:**
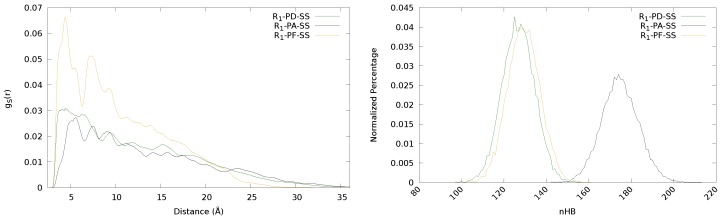
Radial distribution functions (**left**) and normalized percentage of hydrogen bonds (**right**) of the urea-substituted diphenyl (PD)-, diphenyl amino (PA) and phenyl free (PF)-disulfide derivatives.

In order to analyze the effect of the backbone composition, the PD-, PA- and PF-SS disulfides substituted with the urea moiety (R${}_{1}$) were chosen. The results are collected in Table 3. It is observed that HB${}_{tot}$ is similar in PD and PF but is notably increased in the PA derivatives. This result is consistent with the incorporation of an extra amino group that is able to establish hydrogen bonds. This feature is also clearly noticed in Figure 6 (right), where the normalized percentage of hydrogen bonds is represented.

In Figure 6 (left), the radial distribution functions of sulfur for the three derivatives are represented. It can be seen that the PF derivative present higher peaks (larger values of disulfide population) at rather close distances and decay more rapidly than the other two. Taking into account the structure of this derivative (see Figure 1), the absence of the phenyl ring may favour the presence of neighbouring disulfides closer. The steric hindering caused by the phenyls is also clearly seen in the radial distribution functions of the PD and PA, as they show lower values than PF of disulfide population at distances where the phenyl rings are expected to be (<10 Å). Moreover, since in PF and PA the disulfide bond is directly attached to a group that can form hydrogen bonds (the amino group), these interactions may contribute to limiting the mobility of the disulfides and, therefore, hindering their approach. This feature is reflected in the radial distribution functions, where PF and PA show the highest peaks shifted to longer distances compared to that of PD, especially for PA, for which both the reduced mobility due to hydrogen bonding and the steric hindrance of the phenyls contribute to shifting this peak to even longer distances than for PF.

Regarding ω, as it has been previously observed, the main factor is the hydrogen bonding. Formoso et al. showed that both an excessively large or small amount of hydrogen bonds is detrimental for an optimal ω value [52]. Thus, the remarkable increment of hydrogen bonds in the PA derivative with respect to PD and PF (see Table 3), leads to a lower value of ω, which can be related to too rigid chains. Both PD and PF show similar values of ω, which may be explained due to the lack of steric hindrance of the phenyl in PF compensating the less mobility of the sulfurs induced by the hydrogen bonds formed by the extra amino group.

### 3.2. Influence of the Substituents (R${}_{1}$-R${}_{5}$) and Chalcogenide Atoms (X)

In this subsection, the effect of the chemical structure of the polymeric chain (R${}_{n}$) and dichalcogenide bond (S-S vs. Se-Se) is analyzed. Concretely, R${}_{n}$-PD-XX systems have been chosen to carry out such analysis, being *n* = 1–5 and X = S, Se. All the obtained data are collected in Table 4.

**Figure 7:**
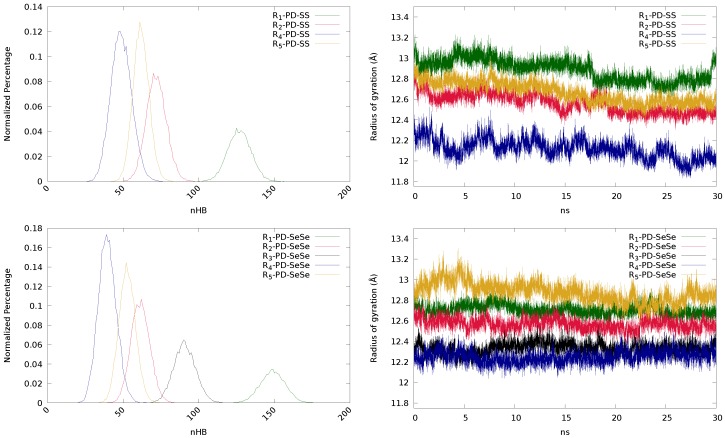
Normalized percentage of hydrogen bonds (**left**) and radius of gyration (**right**), in Å, of the disulfide (**top**) and diselenide (**bottom**) derivatives.

First, we focus on the influence of R${}_{n}$. The differences between chains arise from their distinct ability to establish hydrogen bonds, independently of the nature of the X-X bond. This is clearly seen in Figure 7 (top left), where the normalized percentage of hydrogen bonds is represented for each system. The smallest numbers of HB are provided by the ether- and ester-containing chains ($R_{4}$ and $R_{5}$, respectively), while urea ($R_{1}$) shows the highest number, as expected, due to the presence of the –NH– group. This reduction of HBs induces a decrease in the value of ω, which suggests an excessive mobility of the chains. As it was previously explained, a too small number of hydrogen bonds may be detrimental for the self-healing processes, since too mobile chains may reduce the number of disulfides in the reacting region ($I_{i}$ of the radial distribution function), and therefore, decreasing the value of ω. Inspecting the radius of gyration (Figure 7, top right), chains with larger number of atoms present larger values of radius of gyration, as expected.

Finally, we have analyzed the effect of changing sulfur by selenium, since it has been recently found that selenium can enhance the self-healing process [45,46,47,48,49]. Regarding the number of hydrogen bonds, the same trend as for disulfides can be observed, since the ability to establish HBs is, in principle, independent from the dynamic bond. Nevertheless, with the exception of the urea-containing systems (R${}_{1}$), the diselenide derivatives establish less hydrogen bonds than the disulfides, which pinpoints to different structural reorganizations that may affect to the interaction among functional groups and, therefore, an indirect influence of the dynamic bond might be devised. The substitution of sulfur by selenium also has an impact on the radius of gyration of the systems. Inspecting Figure 7 (top-right and bottom-right panels), the radius of gyration of the systems including diselenide bonds show, in general, smaller variations, indicating a more rigid character than their disulfide counterparts. This could be a consequence of Se being heavier and less mobile than S.

This lower mobility of the Se-containing chains may explain the observed variations in the hydrogen bonding and in ω. For those chains with a large number of HBs, such as the urea-based system (R${}_{1}$), the reduced mobility of Se may enhance the rigidity if the system, keeping the number of hydrogen bonds and even favoring the formation of new bonds. Thus, HB${}_{max}$(R${}_{1}$-PD-SS) = 160, while for the corresponding diselenide is increased to 184. On the other hand, if the molecular chain contains few groups able to create hydrogen bonds, the presence of Se atoms may further limit the capacity to establish hydrogen bonds, since the probability of the functional groups to approach will be smaller (due to lower mobility). As a consequence, the diselenide derivatives show smaller HB${}_{max}$ values. Nevertheless, it is difficult to establish a direct comparison between diselenides and disulfides in terms of ω, since the definition of the regions that characterize this parameter is different. The reacting region ($I_{i}$) for disulfides included all sulfurs laying closer than 4.5 Å, as quantum calculations showed that at this distance the exchange reaction may take place [50]. Nevertheless, 4.25 Å was found to be the distance at which the exchange reaction could be triggered in the case of diselenides [47]. Besides, since diselenides are less mobile, the neighboring region ($I_{ii}$), where diselenides have a non-zero probability to undergo the reaction, has been reduced from 20 to 15 Å. Therefore, the trend observed in Table 4 may not be considered as a general trend.

### 3.3. Theoretical Self-Healing Capacity

As it was mentioned in the Introduction, the theoretical self-healing capacity (at the microscopic level) of a dichalcogenide-based polymer can be estimated by three parameters: the probability to generate radicals by cleavage of the dichalcogenide bond (ρ), the exchange reaction constant (*k*) and the mobility of the chains (ω) [52]. The first two parameters, ρ and *k*, have been calculated in a recent work for a set of disulfide- and diselenide-containing materials [47]. In Table 5, these values have been collected together with those of ω calculated in this work. In addition to *k*, the Gibbs free energy of the transition states ($\Delta G^{TS}$) are given as well. The calculation of the rate constant, using the Eyring equation, requires two main parameters; the activation Gibbs free energy and the pre-exponential factor:

(2) $$k\left(T\right)=Ae^{-\Delta G^{TS}/RT}$$

While the activation energy can be calculated with high accuracy, the calculation of the pre-exponential factor (*A*), which depends on the molecular partition functions, is very challenging [60]. Hence, in order to estimate the theoretical self-healing capacity, $\Delta G^{TS}$ will be used instead of *k*. Since there is no experimental evidence to assess the relative weight of these parameters in the self-healing process, we can only estimate the theoretical self-healing capacity by inspecting the trends of each parameter. Thus, we expect the self-healing capacity to be enhanced for larger values of ρ and ω, and lower values of $\Delta G^{TS}$.

Inspecting Table 5, it is observed that the diselenide compounds have notably smaller reaction barriers than the disulfides. In addition to this, ω is generally larger, with the exception of R${}_{1}$-PD-SS. As a consequence, we would expect larger values of self-healing capacity for diselenides, as it may be for R${}_{1}$-PD-SS, given that it shows the largest value of ω among the PD derivatives.

The effect of the backbone can be analyzed comparing the R${}_{1}$ derivatives for PD, PA and PF. It is observed a remarkably lower reaction barrier for PD due to the stability of the TS produced by the π-π interactions among phenyl rings. These interactions are less effective when the amino group is inserted between the SS bond and the ring in the PA derivative (see Figure 1), so that the barrier is notably increased. The largest ω value is calculated for the PF derivative, but it also shows the highest reaction barrier. Considering that the slight increase of ω after substituting PD by PF may not be enough to compensate the notably larger reaction barrier calculated for PF, the combination of these two factors may suggest that the theoretical self-healing capacity of PF will be lower than that of PD. Moreover, the low values of ω combined with the high reaction barriers shown by the PA derivative suggest that the self-healing capacity of the PA derivate will also be low, in spite of the large value calculated for ρ. As a summary, we may suggest that the largest self-healing capacity corresponds to the PD derivatives.

Finally, comparing the different substituents (R${}_{i}$), similar reaction barriers are observed, between 10 and 12 kcal/mol (except for R${}_{5}$), as well as similar values of ρ. This means that the most important effect is the mobility of the chains (ω). We have found that a smaller number of hydrogen bonds has as a consequence lower values of ω due to too mobile chains (see Table 4) and, thus, the largest self-healing capacity would be expected for R${}_{1}$, the urea derivative, which shows both a low reaction barrier and a notable number of hydrogen bonds among chains.

## 4. Conclusions

In this work, a set of dichalcogenides (including disulfide and diselenide bonds) with different backbones (PA, PD and PF) combined with five different functional groups as substituents have been studied by means of molecular dynamics simulations. First of all, a theoretical protocol is established for creating an adequate model by assessing the relevance of four different parameters, namely, initial guess, chain length, simulation box and simulation time, and then it has been compared to the previous one.

With this new protocol we have calculated different properties of the systems, such as the hydrogen bonding and the mobility of the chains (ω). It has been shown that the backbone has a direct influence in ω, with PD and PF showing similar values, while for PA derivatives a clear decrease is observed. The number of hydrogen bonds established affects the value of ω, in such a way that those systems with an intermediate number of hydrogen bonds are the ones showing larger ω values. Moreover, it can be seen that systems including diselenide bonds tend to show bigger values of ω than their disulfide counterparts (excepting R${}_{1}$-PD-SS).

After the calculation of ω, we combine this parameter with ρ (the probability of radicals to be formed) and $\Delta G^{TS}$ (the reaction barrier of exchange reaction), that were calculated in previous works, to estimate the theoretical self-healing capacity of the systems studied, that will be directly proportional to ρ and ω, and inversely proportional to $\Delta G^{TS}$. We conclude that, in general, the PD-SeSe derivatives show the best theoretical self-healing capacities.

## Figures and Tables

**Table 1:** Total number of hydrogen bonds (HB${}_{tot}$), maximum (HB${}_{max}$), average (HB${}_{ave}$) and minimum (HB${}_{min}$) number of hydrogen bonds at a given step, and ω obtained for the periodic and random starting guess, with and without the extra methyl group in the molecular model (long and short chains, respectively), for 10 and 30 ns of simulation time.

Chain	Guess	HB${}_{tot}$	HB${}_{max}$	HB${}_{ave}$	HB${}_{min}$	ω	HB${}_{tot}$	HB${}_{max}$	HB${}_{ave}$	HB${}_{min}$	ω
		**10 ns**					**30 ns**				
Short	Periodic	746,461	176	149	121	0.0884	2,215,568	178	148	117	0.0776
	Random	730,119	172	146	118	0.0696	2,233,776	184	149	117	0.0627
Long	Periodic	707,860	169	142	115	0.0931	2,184,978	179	146	116	0.0970
	Random	595,587	147	119	91	0.0393	1,917,000	157	128	100	0.0188

**Table 2:** Total number of hydrogen bonds (HB${}_{tot}$), maximum (HB${}_{max}$), average (HB${}_{ave}$) and minimum (HB${}_{min}$) number of hydrogen bonds at a given step, and ω, for three simulation boxes using the random system with long chains after 30 ns of simulation.

Box	HB${}_{tot}$	HB${}_{max}$	HB${}_{ave}$	HB${}_{min}$	ω
40 × 30 × 30	1,917,000	157	128	100	0.0188
40 × 40 × 40	1,900,841	158	127	95	0.0490
50 × 50 × 50	1,902,463	160	127	97	0.1058

**Table 3:** Total number of hydrogen bonds (HB${}_{tot}$), maximum (HB${}_{max}$), average (HB${}_{ave}$) and minimum (HB${}_{min}$) number of hydrogen bonds at a given step, and ω obtained for urea-substituted (R${}_{1}$) disulfides (X = S) including the three backbones PD, PA and PF.

X	R	Backbone	HB${}_{tot}$	HB${}_{max}$	HB${}_{ave}$	HB${}_{min}$	ω
S	R${}_{1}$	PD	1,902,463	160	127	97	0.1058
		PA	2,620,182	213	175	142	0.0419
		PF	1,940,357	161	129	102	0.1224

**Table 4:** Total number of hydrogen bonds (HB${}_{tot}$), maximum (HB${}_{max}$), average (HB${}_{ave}$) and minimum (HB${}_{min}$) number of hydrogen bonds at a given step, and ω obtained for the PD-SS and PD-SeSe systems with different polymeric chains built using the following functional groups: urea (R${}_{1}$), urethane (R${}_{2}$), secondary alcohol (R${}_{3}$), ether (R${}_{4}$) and ester (R${}_{5}$).

		S-S					Se-Se				
Backbone	R	HB${}_{tot}$	HB${}_{max}$	HB${}_{ave}$	HB${}_{min}$	ω	HB${}_{tot}$	HB${}_{max}$	HB${}_{ave}$	HB${}_{min}$	ω
PD	R${}_{1}$	1,902,463	160	127	97	0.1058	2,233,772	184	149	115	0.0418
	R${}_{2}$	1,078,729	100	72	50	0.0471	921,219	87	61	37	0.0538
	R${}_{3}$	—	—	—	—	—	1,359,569	119	91	64	0.0307
	R${}_{4}$	731,072	76	49	26	0.0212	592,646	66	40	16	0.0332
	R${}_{5}$	927,209	83	62	42	0.0348	790,616	74	53	34	0.0443

**Table 5:** Probability to generate sulfenyl and selenyl radicals (ρ), Gibbs free energy of the transition state ($\Delta G^{TS}$), in kcal/mol, rate constant (*k*) at T = 298.15 K, in s${}^{-1}$, and ratio of dichalcogenides in the reaction region (ω) for the PD-SS and PD-SeSe systems with different functional groups, namely, urea (R${}_{1}$), urethane (R${}_{2}$), secondary alcohol (R${}_{3}$), ether (R${}_{4}$) and ester (R${}_{5}$).

		S-S				Se-Se			
Backbone	R	ρ	$\Delta G^{TS}$	k	ω	ρ	$\Delta G^{TS}$	k	ω
PD	R${}_{1}$	0.0051	11.03	4.97·10${}^{4}$	0.1058	0.0122	8.97	1.62·10${}^{6}$	0.0418
	R${}_{2}$	0.0010	12.21	6.78·10${}^{3}$	0.0471	0.0040	8.03	7.95·10${}^{6}$	0.0538
	R${}_{3}$	0.0310	10.46	1.32·10${}^{5}$	—	0.0094	5.99	2.52·10${}^{8}$	0.0307
	R${}_{4}$	0.0101	10.98	5.40·10${}^{4}$	0.0212	0.0041	7.89	1.01·10${}^{7}$	0.0332
	R${}_{5}$	0.0045	16.39	0.58·10${}^{1}$	0.0348	0.0000	7.96	8.98·10${}^{6}$	0.0443
PA	R${}_{1}$	0.2382	18.26	2.46·10${}^{-1}$	0.0419				
PF	R${}_{1}$	0.0682	18.49	1.66·10${}^{-1}$	0.1224				

## Abbreviations

The following abbreviations are used in this manuscript:

MD	Molecular dynamics
RESP	Restrained electrostatic potential
HB	Hydrogen bond
